# 2179. Clinical Characteristics And Outcomes of Peritoneal Dialysis-Related Peritonitis Infection by Ampc β-lactamase-producing Enterobacterales

**DOI:** 10.1093/ofid/ofad500.1801

**Published:** 2023-11-27

**Authors:** Alberto Ordinola Navarro, Jazmín Itzayana Salazar-Leal, Bruno Ali Lopez Luis

**Affiliations:** Instituto Nacional de Ciencias Médicas y Nutrición Salvador Zubirán, Mexico, Distrito Federal, Mexico; Centro Médico Nacional 20 de Noviembre, mexico, Distrito Federal, Mexico; Centro Médico Nacional 20 de Noviembre, mexico, Distrito Federal, Mexico

## Abstract

**Background:**

Peritonitis is one of the most significant complications in patients undergoing peritoneal dialysis (PD). Even when appropriately treated, approximately 20% of all episodes are refractory to treatment. Enterobacteriaceae represent about 10% of cases of peritonitis associated with PD, gram-negative organisms that have inducible beta-lactamase genes known as AmpC are usually more severe clinically and are associated with worse outcomes, including an increased risk of hospitalization and death.

**Methods:**

A retrospective case series in Centro Médico Nacional 20 de Noviembre in Mexico from 2020-2022. We included hospitalized patients diagnosed with peritonitis who met the following criteria: Cloudy peritoneal effluent, Abdominal pain, white-cell counts higher than 100/μL with at least 50% polymorphonuclear cells and culture of peritoneal effluent with the isolation of AmpC β-lactamase-producing Enterobacterales Identified by Vitek 2 and corroborated by phenotypic tests.

**Results:**

30 episodes of peritonitis associated with PD by AmpC enterobacterales were identified. Clinical characteristics are summarized in **Table1**. Their resistance profiles are reported in **Table2**. Two patients had refractory peritonitis, 3 changed modality to hemodialysis, 2 had repeat peritonitis at 90 days due to Serratia marcescens and required removal. Four patients had a new episode of peritonitis due to another organism, and two died secondary to septic shock **Table3.** Notably, this infection may be related to poor hygiene conditions and poor techniques in replacing peritoneal fluid bags since we found that 71% of our patients acquired the infection at their homes. We found that 58% of patients treated with at least two antibiotics occupying 2 routes of administration (intravenous/intraperitoneal) had a good response.

**Figure:**
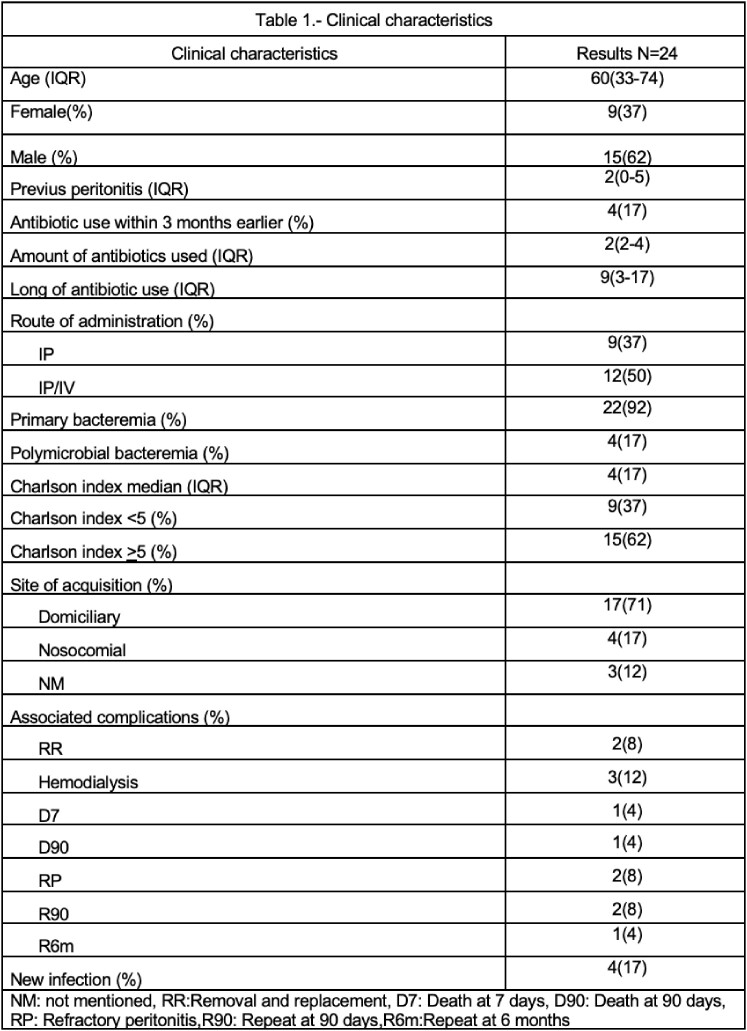

Table 2

**Figure:**
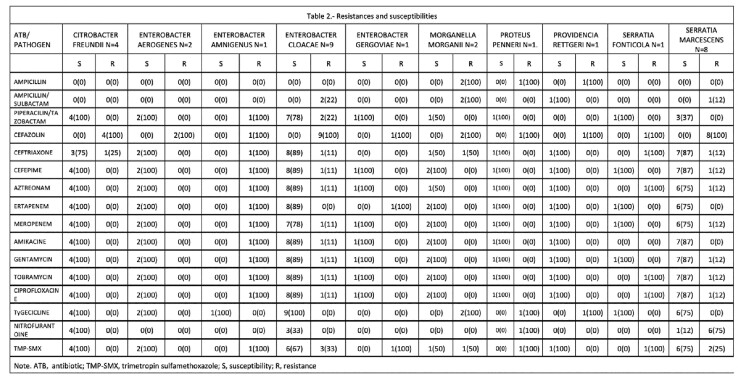

Table3

**Figure:**
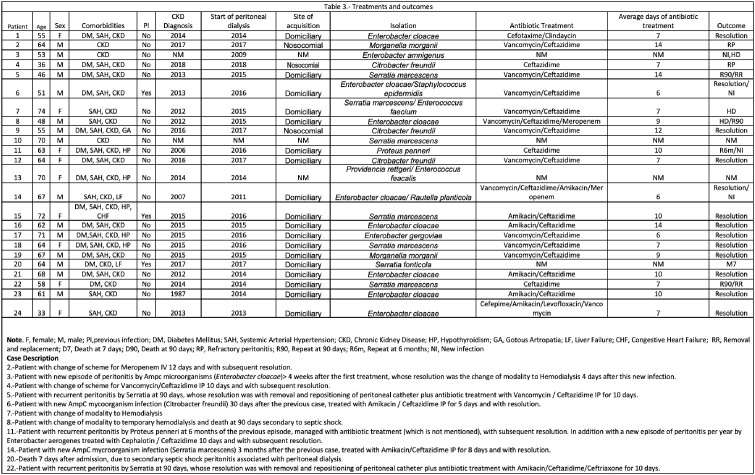

**Conclusion:**

AmpC peritonitis can lead to refractoriness, loss of the catheter, change to hemodialysis and even death, which in our particular context, dual antibiotic therapy may be sufficient; however, removing the catheter improves the response to treatment and decreases refractory peritonitis. The relationship to complications by these agents are high (50% in this study), so aggressive management is necessary for better outcomes in case of isolation.

**Disclosures:**

**All Authors**: No reported disclosures

